# FBLN5 was Regulated by PRDM9, and Promoted Senescence and Osteogenic Differentiation of Human Periodontal Ligament Stem Cells

**DOI:** 10.2174/1574888X18666230822100054

**Published:** 2023-11-29

**Authors:** Mengyao Zhao, Rong Rong, Chen Zhang, Haoqing Yang, Xiao Han, Zhipeng Fan, Ying Zheng, Jianpeng Zhang

**Affiliations:** 1 Laboratory of Molecular Signaling and Stem Cells Therapy, Beijing Key Laboratory of Tooth Regeneration and Function Reconstruction, School of Stomatology, Capital Medical University, Beijing, China;; 2 Department of Endodontics, Beijing Stomatological Hospital, School of Stomatology, Capital Medical University, Beijing, China;; 3 Beijing Laboratory of Oral Health, Capital Medical University, Beijing, China

**Keywords:** Periodontal ligament stem cells (PDLSCs), FBLN5, PRDM9, senescence, osteogenic differentiation, mitogen-activated protein kinase (MAPK), signaling pathway

## Abstract

**Objectives:**

Periodontal ligament stem cells (PDLSCs) are ideal seed cells for periodontal tissue regeneration. Our previous studies have indicated that the histone methyltransferase PRDM9 plays an important role in human periodontal ligament stem cells (hPDLSCs). Whether FBLN5, which is a downstream gene of PRDM9, also has a potential impact on hPDLSCs is still unclear.

**Methods:**

Senescence was assessed using β-galactosidase and Enzyme-linked immunosorbent assay (ELISA). Osteogenic differentiation potential of hPDLSCs was measured through Alkaline phosphatase (ALP) activity assay and Alizarin red detection, while gene expression levels were evaluated using western blot and RT-qPCR analysis.

**Results:**

FBLN5 overexpression promoted the osteogenic differentiation and senescence of hPDLSCs. FBLN5 knockdown inhibited the osteogenic differentiation and senescence of hPDLSCs. Knockdown of PRDM9 decreased the expression of FBLN5 in hPDLSCs and inhibited senescence of hPDLSCs. Additionally, both FBLN5 and PRDM9 promoted the expression of phosphorylated p38 MAPK, Erk1/2 and JNK. The p38 MAPK pathway inhibitor SB203580 and the Erk1/2 pathway inhibitor PD98059 have the same effects on inhibiting the osteogenic differentiation and senescence of hPDLSCs. The JNK pathway inhibitor SP600125 reduced the senescence of hPDLSCs.

**Conclusion:**

FBLN5 promoted senescence and osteogenic differentiation of hPDLSCs *via* activation of the MAPK signaling pathway. FBLN5 was positively targeted by PRDM9, which also activated the MAPK signaling pathway.

## 1 INTRODUCTION

Periodontitis is a progressive destruction of periodontal tissues mainly caused by plaque accumulation. Periodontitis is the primary factor in tooth loss and it becomes more prevalent with age. Although current treatment methods of periodontitis include microbial pathogens elimination and biomaterials have been used in treating bone defects, these treatments offer limited benefits for the regeneration of periodontal tissue. Hence, periodontal researchers face a significant challenge in achieving the full regeneration of complex periodontal tissue, including cementum, periodontal membrane, and alveolar bone. Fortunately, the application of mesenchymal stem cells will be useful for periodontal tissue regeneration due to their multipotency [1, 2].

Periodontal ligament stem cells (PDLSCs) are a type of mesenchymal stem cells that are derived from the periodontal membrane. These cells possess several key characteristics including their ability to differentiate into multiple cell types and exhibit high proliferation rates *in vitro* and *in vivo* [3]. PDLSCs were then widely used for the studies of periodontal tissue regeneration. Co-transplantation of human or rat PDLSCs with hydroxyapatite scaffolds into artificial periodontal defects in mandibular molars of immunodeficient mice or dorsal submuscular of rats was subsequently confirmed by the reconstruction of bone, cementum-like, and periodontal ligament-like structures at the transplantation sites [4-6]. In a randomized controlled trial comparing 28 patients with periodontitis [7], patients who underwent autologous PDLSCs and open flap debridement had a more significant increase in bone density in the defect area at 3-12 months compared to those treated with open flap debridement only. The findings highlight the potential clinical applications of PDLSCs in periodontal tissue engineering. The discovery and application of PDLSCs have opened up new avenues for regenerative therapy of periodontal tissue abnormalities caused by periodontitis. However, the highly proliferative and differentiated characteristics of PDLSCs tend to decrease with aging [8]. Studies have shown that PDLSCs have reduced differentiation potential, increased apoptosis, and decreased immunosuppressive capacity with aging [9-11]. Wu *et al*. [12] found that the ability of PDLSCs to form cell sheets and osteogenic differentiation decreased with aging by inducing PDLSCs with a sheet induction medium. To improve the differentiation and regenerative function of PDLSCs, it is necessary to gain a detailed understanding of their molecular regulatory mechanism.

Epigenetic inheritance, which refers to the transmission of heritable changes in traits without alterations to DNA base sequences, is an important driver of stem cell senescence. Histone modifications, such as histone methylation and acetylation, are among the most intensively studied markers of cellular senescence [13]. The PRDM family is characterized by its PR-SET structural domain, which regulates a variety of biological processes including proliferation, differentiation, cell cycle progression, and intracellular homeostasis maintenance in immune cells through intrinsic histone methyltransferase activity or interaction with other nuclear chromatin-modifying enzymes [14]. PRDM9 is a PRDM family member with intrinsic methyltransferase activity that modifies chromosome structure *via* trimethylation of histone H3 lysine4 and 36. Recently, studies by our group have revealed that knocking down the PRDM9 gene in hPDLSCs leads to an increase in migratory and chemotaxis abilities, as well as a decrease in osteogenic differentiation ability [15, 16]. These findings indicated that PRDM9 may play an important role in promoting the regeneration of hPDLSCs into periodontal tissue, but how PRDM9 works requires further study.

According to our previous study results, gene microarray showed that FBLN5 is a downstream gene of PRDM9 [15]. FBLN5 is a cellular matrix protein that promotes elastin production [17]. It has been found to regulate cell growth, migration, tissue repair, and tumorigenesis [18, 19]. Studies have shown that FBLN5 is a very sensitive marker of aging. mRNA and protein levels of FBLN5 were drastically decreased in fibroblasts from aged donors and controlled redox homeostasis simultaneously [20]. FBLN5 also played an important role in suppressing inflammatory processes. It is lowly expressed in cartilage tissues of patients with osteoarthritis. Overexpression of FBLN5 reduced IL-1β-induced inflammation in chondrocytes [21] and also reduced the inflammatory expression of skin after burns [22]. In addition, this gene has been associated with photoaging skin [23] and age-related macular degeneration [24]. However, it is unclear whether FBLN5 has a regulatory function in hPDLSCs.

In this research, we aim to investigate whether FBLN5 plays a regulatory role in senescence and osteogenic differentiation of hPDLSCs and to identify the underlying regulatory mechanisms. The findings of this study could provide potential targets for promoting periodontal regeneration.

## 2 MATERIALS AND METHODS

### 2.1 Cell Cultures

The hPDLSCs were extracted from orthodontic teeth (aged 18-25 years) obtained with informed consent from patients in accordance with the rules of Beijing Stomatological Hospital, Capital Medical University. (Ethics Review Number: CMUSH-IRB-KJ-PJ-2022-33).

Periodontal ligament tissue was scraped from the mid-roots and washed in sterile PBS containing 1% penicillin and streptomycin, digested in a 1:1 mixture of I Collagenase (3 mg/ml) and Dispase (4 mg/ml) at 37°C for 30 mins, and the cells were collected by centrifugation. Cells were resuspended in α-MEM medium (containing 15% fetal bovine serum, 100 U/ml penicillin, 100 μg/ml streptomycin, 2 mmol/L glutamine) and inoculated in culture dishes at 37°C and 5% CO_2_.

### 2.2 Plasmid Construction and Viral Infection

GenePharma in China provided the PRDM9 shRNA, the FBLN5 shRNA, and the control shRNA lentivirus. The PRDM9 shRNA sequence used in our study is 5'-GAGTGACAGCGTAACACC-3'PDLSCs. The FBLN5 shRNA sequence used in this study is 5'- GGATCCTATTCTTGTACATGC-3'. The control shRNA (Consh) targeted sequence is 5'-TTCTCCGAACGTGTCACGTTTC‐3'. Human full-length FBLN5 complementary cDNA was synthesized through a gene synthesis method and subcloned into LV5 retroviral vector which was obtained from GenePharma, China. After 12 hours for virus transfected, hPDLSCs were cultured in a complete medium containing 6 μg/ml polybrene. Cells were screened for 3 days after 48 hours with 1 μg/ml puromycin.

### 2.3 β-galactosidase Staining (β-gal)

The expression of β-galactosidase in hPDLSCs was detected according to the kit operating instructions (Cat No: GMS 10012. 1, genemed, China). hPDLSCs that inoculated at 2×10^4^ per well in 24-well plates, washed, fixed, and stained, followed by overnight incubation at 37°C. The cells were then observed under a light microscope, and the number of β-galactosidase staining-positive cells was counted using the Image J program.

### 2.4 Enzyme Linked Immunosorbent Assay (ELISA)

The cells were lysed and total protein was extracted using RIPA lysis buffer and protein quantification was performed using the Bradford method. Sample at 250 μg/100 μl per well were added according to the kit operating instructions (Catalog No.CSB-EL023391HU, CUSABIO, China), and each sample was repeated three times. The samples were then incubated at 37°C for 2h. Following this, 100 μl of biotin antibody working solution, 100 μl of HRP affinity, and 90 μl of TMB substrate were added sequentially. Finally, each well-received 50 μl of stop solution and the absorbance of the cell cultures was measured at 450 nm on a multi-plate reader within 5 minutes.

### 2.5 Western Blot

SDS polyacrylamide gel electrophoresis tests were performed as the previous study [25]. Protein expression levels were detected by ECL luminescence imaging. The primary antibodies used in this experiment included anti-PRDM9(PA541161;Invitrogen); anti-FBLN5(Catalog # 3095-FB; R&D system); anti-p16 (Cat No. 10883-1-AP;proteintech), anti-p53(Cat No. 60283-2-Ig; proteintech), anti-phospho-p38 MAPK(Cat No. 28796-1-AP ;Proteintech), anti-p38MAPK(Cat No. 14064-1-AP ;Proteintech), anti-phospho-Erk1/2(Cat No: 80031-1-RR;Proteintech), anti-Erk1/2(CatNo. 11257-1-AP; Proteintech); anti-phospho-JNK (CatNo. 4668; Cell Signaling Technology), anti‐JNK (Cat No. 9258; Cell Signaling Technology) and anti-GAPDH (Cat No. 60004-1-1g; Proteintech)

### 2.6 Alkaline Phosphatase (ALP) Activity Assay and Alizarin Red Detection

Osteogenic induction medium with 100 M/mL ascorbic acid, 2 mM -glycerophosphate, 1.8 mM KH2PO4, and 10 nM dexamethasone was used to cultivate hPDLSCs. ALP activity assay and Alizarin red detection were performed according to the previous studies [26].

### 2.7 Reverse Transcriptase Polymerase Chain Reaction (RT-PCR) and Real-time RT-PCR

Total RNA was extracted using Invitrogen Trizol reagents, and 2 μg of each sample was uploaded for reverse transcription. The QuantiTect SYBR Green PCR kit (Qiagen) and an icycler iQ Multicolor Realtime RTPCR Detection System were used for real-time RTPCR reactions. The GPADH primers were as follows: Forward primer: 5'-AGGTCGGTGTGAACGGATTTG-3'; Reverse primer: 5'-TGTAGACCATGTAGTTGAGGTCA-3'. The FBLN5 primers were as follow: Forward primer: 5'- CAATTTACAAGGGGGCTTCA-3'; Reverse primer: 5'-GGGTTCTCAGCAGGACACAG-3'.

### 2.8 Statistical Analysis

All the statistical data in this experiment were analyzed using GraphPad Prism (version 8.2.1) software. One-way ANOVA and student’s t-test were used to determine statistical significance. *P* < 0.05 was considered statistically significant.

## 3 RESULTS

### 3.1 Overexpression of FBLN5 Promoted the Osteogenic Differentiation and Senescence of hPDLSCs

**Fig. (1):**
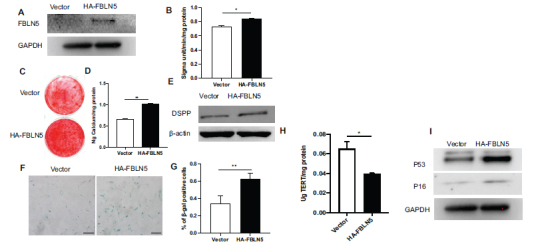
Overexpression of FBLN5 up-regulated the osteogenic differentiation ability and senescence of hPDLSCs. (**A**) Western-blot analysis revealed that FBLN5 was overexpressed in hPDLSCs. GAPDH was used as an internal control; (**B**) ALP activity assay; (**C**) Alizarin Red staining. (**D**) Calcium quantification; (**E**) Western-blot demonstrated DSPP expression during hPDLSCs’ osteogenic differentiation; (**F**, **G**) β-gal staining; (**H**) ELISA results revealed hPDLSCs’ telomerase reverse transcriptase expression. (**I**) Western-blot analysis revealed that the presence of P16 and P53. As an internal control, GAPDH was used. Student's t-test was used to determine statistical significance. Error bars represent the SD (n = 3), * *P*< 0.05, **P < 0.01; scale bar: 200 μm.

The lentivirus vector carrying the HA-FBLN5 sequence was transduced into hPDLSCs and confirmed by western blot after 3 days of puromycin (1 g/mL) selection (Fig. **1A**). HA-FBLN5 group showed a higher level of ALP activity in hPDLSCs than the control group after 5 days of osteogenesis induction, according to the results of ALP assays (Fig. **1B**). After 14 days of osteogenesis induction, alizarin red staining and calcium quantification results showed that the HA-FBLN5 group had more mineralization compared to the control group (Figs. **1C**, **1D**). Western blot analysis further revealed that DSPP expression was significantly greater in the HA-FBLN5 group compared to the control group (Fig. **1E**). Interestingly, SA-β-gal staining and quantitative analysis results revealed that the HA-FBLN5 group had an increase in SA-β-gal positive cells than the control group (Figs. **1F**, **1G**). ELISA results revealed that FBLN5 overexpression reduced telomerase activity (Fig. **1H**). Additionally, Western blot analysis revealed that the HA-FBLN5 group expressed more P16 and P53 proteins than the control group in hPDLSCs (Fig. **1F**).

### 3.2 Knock-down of FBLN5 Inhibited the Osteogenic Differentiation and Senescence of hPDLSCs

**Fig. (2):**
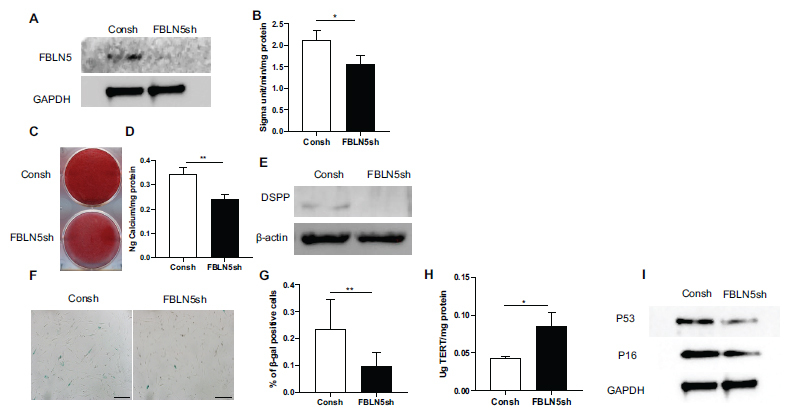
Knock-down FBLN5 down-regulated the osteogenic differentiation ability and senescence of hPDLSCs. (**A**) Western-blot analysis revealed that FBLN5 was efficiently knocked down in hPDLSCs. As an internal control, GAPDH was used; (**B**) ALP activity assay; (**C**) Alizarin Red staining. (**D**) Calcium quantification; (**E**) Western-blot demonstrated DSPP expression during hPDLSCs osteogenic differentiation; (**F**, **G**) β-gal staining; (**H**) ELISA results showed hPDLSCs’ telomerase reverse transcriptase expression; (**I**) Western-blot analysis revealed that the presence of P16 and P53. As an internal control, GAPDH was used. Student's t-test was used to determine statistical significance. Error bars represent the SD (n = 3), *P < 0.05, **P < 0.01; scale bar: 200 μm.

FBLN5 shRNA lentivirus was transfected into hPDLSCs and after 3 days of puromycin (1 μg/mL) selection, the knock-down efficiency of FBLN5(FBLN5sh) was evaluated using western blot (Fig. **2A**). The FBLN5sh group showed a lower level of ALP activity in hPDLSCs than the control group after 5 days of osteogenesis induction, according to the results of ALP assays (Fig. **2B**). Two weeks after osteogenesis induction, alizarin red staining and calcium quantification revealed that the FBLN5sh group had less mineralization than the control group (Figs. **2C**, **2D**). Western blot analysis revealed the expression of DSPP was significantly lower in the FBLN5sh group than the control group (Fig. **2E**). SA-β-gal staining and quantitative analysis results revealed that the FBLN5sh group had a decrease of SA-β-gal positive cells than the control group (Figs. **2F**, **2G**). ELISA results revealed that knock-down of FBLN5 increased telomerase activity (Fig. **2H**). Western blot analysis revealed that the FBLN5sh group expressed fewer P16 and P53 proteins than the control group in hPDLSCs (Fig. **2I**).

### 3.3 Knock-down of PRDM9 Decreased the Expression of FBLN5 and Inhibited Senescence in hPDLSCs

**Fig. (3):**
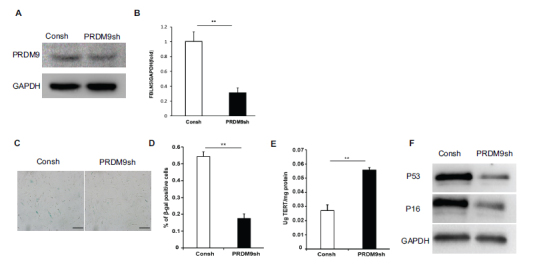
Knockdown PRDM9 promoted the senescence of hPDLSCs. (**A**) Western-blot analysis revealed that PRDM9 was efficiently knocked down in hPDLSCs. As an internal control, GAPDH was used; (**B**) RT-qPCR results showed the expression of FBLN5 in hPDLSCs. GAPDH was used as an internal control. (**C**, **D**) β-gal staining; (**E**) ELISA results showed hPDLSCs’ telomerase reverse transcriptase; (**F**) Western-blot analysis revealed that the presence of P16 and P53. As an internal control, GAPDH was used. Student's t-test was used to determine statistical significance. Error bars represent the SD (n = 3); *P < 0.05, **P < 0.01, scale bar: 200 μm.

PRDM9 shRNA lentivirus was transfected into hPDLSCs and after 3 days of puromycin (1 μg/mL) selection, the knock-down efficiency of PRDM9(PRDM9sh) was evaluated using western blot (Fig. **3A**). The RT-qPCR results confirmed that FBLN5 expression was reduced after PRDM9 knockdown (Fig. **3B**). The SA-β-gal staining and quantitative analysis results showed a decrease in the number of SA-β-gal positive cells in the PRDM9 knockdown group (Figs. **3C**, **3D**). The ELISA results reviewed that knock-down of PRDM9 increased telomerase activity (Fig. **3E**). Additionally, western blot analysis revealed that the PRDM9sh group expressed fewer P16 and P53 proteins than the control group in hPDLSCs (Fig. **3F**).

### 3.4 FBLN5 and PRDM9 Promoted the Expression of Phosphorylated p38 MAPK, Erk1/2, and JNK in hPDLSCs

**Fig. (4):**
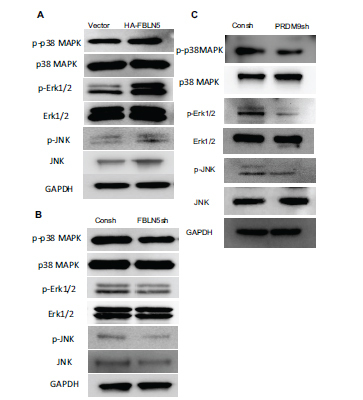
FBLN5 and PRDM9 promoted MAPK signaling pathways expression in hPDLSCs. (**A**) When compared to the control group, phosphorylated p38 MAPK, Erk1/2, and JNK, as well as p38 MAPK, Erk1/2, and JNK, were expressed in HA-FBLN5 hPDLSCs. GAPDH was used as an internal control. (**B**) Western blot analysis revealed that phosphorylated p38 MAPK, Erk1/2 and JNK, as well as p38 MAPK, Erk1/2, JNK, were expressed in FBLN5sh hPDLSCs compared to the control group. As an internal control, GAPDH was used. (**C**) Western blot analysis revealed that phosphorylated p38 MAPK, Erk1/2 and JNK, as well as p38 MAPK, Erk1/2, JNK, were expressed in PRDM9sh hPDLSCs compared to the control group. An internal control, GAPDH was used as an internal control.

The expression of phosphorylated p38 MAPK, p38 MAPK, phosphorylated Erk1/2, Erk1/2, phosphorylated JNK, and JNK were all identified using western blot. The results showed that the HA-FBLN5 group displayed increased phosphorylation of p38 MAPK, Erk1/2, and JNK, while the expression of p38 MAPK, Erk1/2, and JNK remained unchanged (Fig. **4A**). Conversely, the FBLN5sh group displayed decreased phosphorylation of p38 MAPK, Erk1/2, and JNK, with no change in expression of p38 MAPK, Erk1/2, and JNK (Fig. **4B**). Furthermore, the PRDM9sh group showed reduced expression of phosphorylated p38 MAPK, Erk1/2, and JNK, while the expression of p38 MAPK, Erk1/2, and JNK remained unchanged (Fig. **4C**).

### 3.5 P38 MAPK, and Erk1/2 Pathways Inhibitors Repressed the Phosphorylation of p38 MAPK, Erk1/2 Separately and Inhibited Senescence and Osteogenic Differentiation in hPDLSCs Activated by FBLN5

**Fig. (5):**
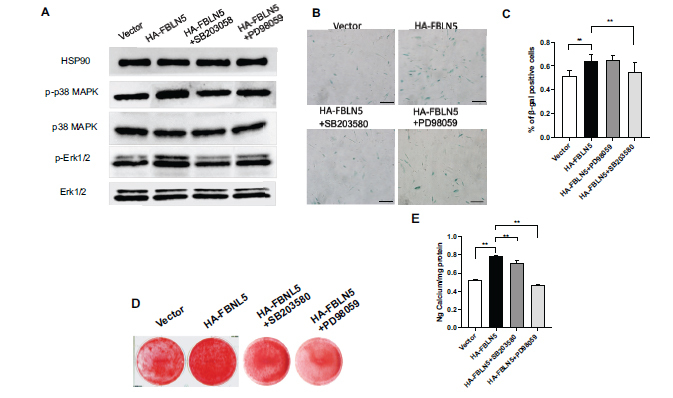
P38 MAPK, Erk1/2 pathways inhibitors depressed aging and osteogenic differentiation of hPDLSCs enhanced by FBLN5. (**A**) 20 μM SB203580 and 10 μM PD98059 were used to treat HA-FBLN5 hPDLSCs respectively for 2 hours. The expression of phosphorylated p38 MAPK, Erk1/2, as well as p38 MAPK, Erk1/2 in hhPDLSCs demonstrated by western blot. As an internal control, HSP90 was utilized. (**B**, **C**) β-gal staining; (**D**) Alizarin Red staining; (**E**) Calcium quantification. One-way ANOVA was used to determine the statistical significance. Error bars represent the SD (n = 3), **P < 0.01, scale bar: 200 μm.

We used specific inhibitors including 20 μM SB203580 and 10 μM PD98059 to block p38 MAPK, Erk1/2 pathways separately for 2 hours. Based on the western blot results, HA+SB203580 group and HA+PD98059 group showed a decrease in the phosphorylation of p38 MAPK and Erk1/2 that were activated by FBLN5, while p38 MAPK and Erk1/2 expression remained unchanged (Fig. **5A**). SB203580, and PD98059 significantly reduced the senescence of hPDLSCs promoted by FBLN5, according to the results of SA-β-gal and quantitative analysis (Figs. **5B**, **5C**). The pathway inhibitors significantly reduced the FBLN5-activated osteogenic differentiation in hPDLSCs, according to alizarin red staining and quantitative calcium assays (Figs. **5D**, **5E**).

### 3.6 JNK Inhibitor Repressed the Phosphorylation of JNK and Inhibited Senescence of hPDLSCs Activated by FBLN5

**Fig. (6):**
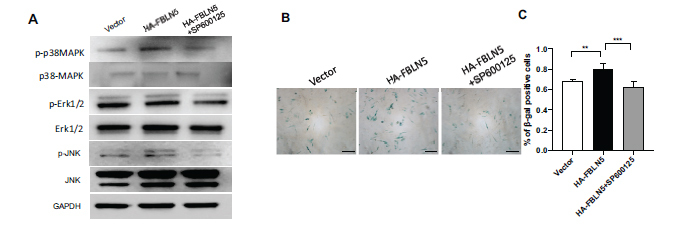
JNK pathway inhibitor depressed on aging of hPDLSCs enhanced by FBLN5. (**A**) Treated HA-FBLN5 hPDLSCs with 20 μM SP600125 for 2 hours. The expression of phosphorylated p38 MAPK, Erk1/2, JNK along with p38 MAPK, Erk1/2, JNK in hPDLSCs were demonstrated by western blot. As an internal control, GAPDH was utilized. (**B**, **C**) β-gal staining. One-way ANOVA was used to determine the statistical significance. Error bars represent the SD (n = 3), **P < 0.01, ***P < 0.001; scale bar: 200 μm.

We used the JNK signaling inhibitor SP600125 for 2 hours to investigate whether it was involved in the FBLN5-enhanced aging of hPDLSCs. According to western blot results, the HA-FBLN5+SP600125 group showed decreased phosphorylation of JNK activated by FBLN5, while JNK expression remained unchanged (Fig. **6A**). Additionally, SP600125 significantly reduced the senescence of hPDLSCs that were promoted by FBLN5, as indicated by the SA-β-gal and quantitative analysis results (Figs. **6B**, **6C**).

## 4 DISCUSSION

Aging of stem cells is regulated by multiple factors, including intrinsic cellular changes such as DNA damage, epigenetic regulation, and external changes in the ecological niche microenvironment. The accumulation of lipofuscin in lysosomes with aging leads to an increase in lysosome volume and number, which further increases the expression of β-galactosidase in lysosomal [27, 28]. Telomeres, one of the three major elements that keep chromosomes intact and stable, are closely related to cellular aging [29]. Telomere shortening in senescent mice causes the accumulation of DNA damage and alteration of gene expression, leading to apoptosis and ultimately affecting stem cell function. The activation of p53, a downstream effector induced by DNA damage, can prevent continuous replication of damaged DNA by inhibiting cell cycle progression and promoting cellular senescence [30], which inhibits the differentiation and self-renewal ability of stem cells but also inhibits tumorigenesis. P16 is repressed in early embryogenesis and is progressively induced during senescence [31]. Thus, β-galactosidase, telomerase reverse transcriptase, p53 and p16 are important markers for detecting cellular senescence and are also served as important targets for delaying stem cell aging. Our results suggest that FBLN5 promotes the expression of β-galactosidase, p53 and p16 and decreases the telomerase reverse transcriptase, which demonstrates that FBLN5 promotes senescence of hPDLSCs. PRDM9 has also been confirmed to promote senescence of hPDLSCs. Our previous findings demonstrated that knockdown of PRDM9 inhibited the osteogenic differentiation of hPDLSCs [16]. The findings of this study validated that knocking down PRDM9 downregulated the expression of FBLN5, which promotes osteogenic differentiation of hPDLSCs. In summary, the regulation of hPDLSCs senescence and osteogenic differentiation by FBLN5 may be positively influenced by the regulation of PRDM9.

MAPK is crucial for biological processes such as individual development, tissue and organ regeneration, and tumor formation. We examined how FBLN5 and PRDM9 regulate hPDLSCs in relation to MAPK signaling pathways. The results showed that both FBLN5 and PRDM9 can promote phosphorylation of JNK, Erk1/2, p38 MAPK. Pathway inhibitors can reduce the phosphorylation of JNK, Erk1/2, p38 MAPK promoted by FBLN5 and PRDM9. Therefore, we can conclude that PRDM9 regulates FBLN5 *via* MAPK signaling pathways to further regulate hPDLSCs.

Several studies have confirmed that the MAPK pathway is critical in regulating cell senescence. For example, targeting p38 MAPK prevented or rescued intestinal villi aging and identified it as an anti-aging target [32]. Mitochondrial morphological abnormalities and dysfunction associated with impaired MAPK/Erk signaling were found in the brains of aged Parkinson's mice mutant for LRRK2 (leucine-rich repeat kinase 2) [33]. In the presence of age-related chronic stress responses, p38 MAPK and SAPK/JNK may exhibit alterations in basal activity levels, and these physiological signals promoted the aging process and the decline of senescent tissues. P38 MAPK and SAPK/JNK were shown to be major signaling pathways promoting the initiation and progression of aging and cardiovascular disease phenotypes [34, 35]. Our results indicate that FBLN5 regulates the aging of hPDLSCs through MAPK signaling pathways. Moreover, inhibition of MAPK signaling pathways suppresses FBLN5‐enhanced senescence of hPDLSCs.

Many studies have also demonstrated that osteogenic differentiation of PDLSCs is related to the MAPK signaling pathway. For instance, CDR1, a miR-7 inhibitor, was found to promote the osteogenic differentiation of PDLSCs by phosphorylating Smad and p38 MAPK [36]. An Erk1/2 specific inhibitor significantly inhibited the Bmi1-induced osteogenic differentiation of PDLSCs [37]. Therefore, we explored the role of the MAPK signaling pathway in FBLN5-mediated regulation of osteogenic differentiation in PDLSCs. Our results showed that inhibitors of p38 MAPK and Erk1/2 suppressed the expression of phosphorylated p38 MAPK, Erk1/2 which were promoted by FBLN5, as well as the mineralization of hPDLSCs. These findings demonstrated that FBLN5 regulates the osteogenic differentiation of hPDLSCs *via* p38 MAPK and Erk1/2 signaling pathways.

## CONCLUSION

Typically, the differentiation ability of cells decreases with aging. The mechanism of differentiation ability may vary depending on the different stimuli [26, 38]. Our results showed that FBLN5, which is positively targeted by PRDM9, promoted the senescence and osteogenic differentiation of hPDLSCs *via* MAPK signaling pathways. PRDM9 also activated the MAPK signaling pathways. These findings may provide potential targets for the biological regulation of PDLSCs and regenerative treatment of periodontal tissues. However, the exact mechanism still needs to be further investigated.

## Data Availability

All data generated or analyzed during this study are included in this article and its supplementary material files. Further inquiries can be directed to the corresponding author.

## LIST OF ABBREVIATIONS

ALP: Alkaline Phosphatase
ELISA: Enzyme Linked Immunosorbent Assay
hPDLSCs: Human Periodontal Ligament Stem Cells
MAPK: Mitogen-activated Protein Kinase
PRDM: PR-SET Domain
β-gal: β-galactosidase Staining
